# Ecological Load and Balancing Selection in Circumboreal Barnacles

**DOI:** 10.1093/molbev/msaa227

**Published:** 2020-09-08

**Authors:** Joaquin C B Nunez, Stephen Rong, Alejandro Damian-Serrano, John T Burley, Rebecca G Elyanow, David A Ferranti, Kimberly B Neil, Henrik Glenner, Magnus Alm Rosenblad, Anders Blomberg, Kerstin Johannesson, David M Rand

**Affiliations:** 1 Department of Ecology and Evolutionary Biology, Brown University, Providence, RI; 2 Center for Computational Molecular Biology, Brown University, Providence, RI; 3 Department of Ecology and Evolutionary Biology, Yale University, New Haven, CT; 4 Institute at Brown for Environment and Society, Brown University, Providence, RI; 5 Department of Biological Sciences, University of Bergen, Bergen, Norway; 6 Department of Chemistry and Molecular Biology, University of Gothenburg, Lundberg Laboratory, Göteborg, Sweden; 7 Department of Marine Sciences, University of Gothenburg, Tjärnö Marine Laboratory, Strömstad, Sweden

**Keywords:** barnacles, *Semibalanus balanoides*, balancing selection, ecological genomics, ecological load

## Abstract

Acorn barnacle adults experience environmental heterogeneity at various spatial scales of their circumboreal habitat, raising the question of how adaptation to high environmental variability is maintained in the face of strong juvenile dispersal and mortality. Here, we show that 4% of genes in the barnacle genome experience balancing selection across the entire range of the species. Many of these genes harbor mutations maintained across 2 My of evolution between the Pacific and Atlantic oceans. These genes are involved in ion regulation, pain reception, and heat tolerance, functions which are essential in highly variable ecosystems. The data also reveal complex population structure within and between basins, driven by the trans-Arctic interchange and the last glaciation. Divergence between Atlantic and Pacific populations is high, foreshadowing the onset of allopatric speciation, and suggesting that balancing selection is strong enough to maintain functional variation for millions of years in the face of complex demography.

## Introduction

The relationship between genetic variation and adaptation to heterogeneous environments remains a central conundrum in evolutionary biology (Botero et al. 2015). Classical models of molecular evolution predict that populations should be locally adapted to maximize fitness (Williams 1966). However, species inhabiting highly heterogeneous environments violate this expectation: If gene flow is high in relation to the scale of environmental heterogeneity, species will harbor variation that is beneficial in one condition but deleterious in another (Gillespie 1973), and the resulting ecological load (i.e., the fitness difference between the best and the average genotype across the range of environments where offspring may settle) will prevent local adaptation. Conversely, if gene flow is low, favored alleles will become locally fixed and species should display low levels of genetic variation. Paradoxically, many natural populations living in variable environments possess high dispersal capabilities and harbor more variation than expected under classical models (Metz and Palumbi 1996; Mackay et al. 2012; Messer and Petrov 2013; Bergland et al. 2014). This disconnect between data and theory has motivated the hypothesis that balancing selection, a process where selection favors multiple beneficial alleles at a given locus, is at play to maintain adaptations in these habitats (Levene 1953; Hedrick 2006).

The northern acorn barnacle (*Semibalanus balanoides*) is a model system to study adaptations to ecological variability. This barnacle is a self-incompatible, simultaneous hermaphrodite which outcrosses only with adjacent individuals. Adult barnacles are fully sessile and occupy broad swaths of intertidal shores in both the North Pacific and North Atlantic oceans. These habitats experience high levels of cyclical and stochastic ecological heterogeneity which impose strong selection at multiple spatial scales: microhabitats (intertidal shores), mesohabitats (bays and estuaries), and macrohabitats (continental seaboards) (Schmidt et al. 2008; Nunez et al. 2020). Barnacle larvae, on the other hand, engage in extensive pelagic dispersal by ocean currents (70–100 km in 5–7 weeks) and may settle in habitats completely different from those of their parents (Flowerdew 1983). This contrast between strong adult selection and high juvenile dispersal prevents local adaptation. In addition, *S. balanoides* has a complex demography. It originated in the Pacific, and colonized the Atlantic during the many waves of the trans-Arctic interchange (1–3 Ma) (Vermeij 1991). Like most circumboreal species, it was subjected to drastic range shifts due to the Pleistocene glacial cycles (Wares and Cunningham 2001; Flight et al. 2012), and more recently due to anthropogenic climate change (Jones et al. 2012). As such, *S. balanoides* is a premier system to study how adaptive genetic variation is maintained over broad spatial and evolutionary scales, in the face of ecological load.

**Fig. 1.**
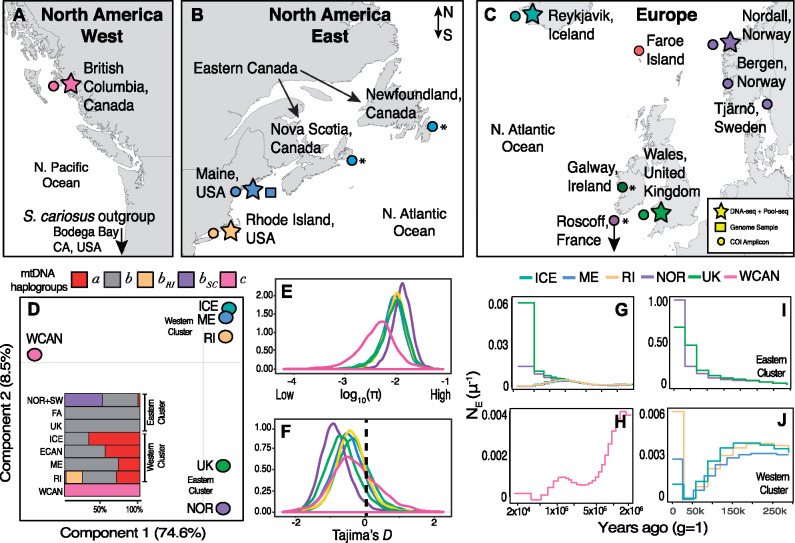
Genetic variation and phylogeography. (*A*) Map of the North Pacific coast of North America with collection sites indicated. (*B*) Collections in the Atlantic Eastern coast of North America. (*C*) Collections in the Atlantic European coast. For (*A*), (*B*), and (*C*), stars indicate sites where a single individual and a pool of multiple individuals were collected, the square indicates the site from which the reference genome was constructed, and the circles indicate sites where *COX I* data were collected. The asterisks indicate cases where *COX I* data were downloaded. (*D*) PCA with pool-seq data from all populations. The colors represent populations: Pacific Canada (WCAN; pink), Maine (ME; blue), Rhode Island (RI; yellow), Iceland (ICE; dark green), Norway (NOR; purple), United Kingdom (UK; light green). (*D*-inset) Distribution of mitochondrial haplotypes across all populations. The names *a*, *b* (including *b*_RI_ and *b*_SC_), and *c* represent common mtDNA haplotypes observed in populations. (*E*) Nucleotide diversity (log_10_*π*) for all nuclear genes across all populations. (*F*) Tajima’s *D* for all nuclear genes across all populations. The dashed vertical line marks 0, the expected value under a neutral model. The *y*-axis in (*E*) and (*F*) shows the density of observations. (*G*) Demographic reconstruction for North Atlantic individuals showing demographic changes from 2 Ma to 200 ka. (*H*) North Pacific individual showing demographic changes from 2 Ma to 200 ka. (*I*) Demographic changes in British and Norwegian individuals. (*J*) Plot of recent (today–250 ka) demographic changes in the North American and Icelandic individuals.

Three decades of work have shown that balancing selection, via marginal overdominance (a case where the harmonic mean fitness of heterozygous genotypes must be larger than that of either homozygote) (Levene 1953), maintains adaptive variation at the metabolic gene Mannose-6-phospate isomerase (*Mpi*) in barnacles across the entire North Atlantic basin (Schmidt and Rand 1999; Dufresne et al. 2002; Rand et al. 2002; Veliz et al. 2004; Nunez et al. 2020). These findings motivate two questions which are addressed in this study. First, how pervasive are balanced polymorphisms in the barnacle genome? And, second, what genes are targets of balancing selection? To investigate functional polymorphism in *S. balanoides*, we quantified genomic variation in North Pacific and North Atlantic populations (fig. 1*A*–*C*). In the Pacific, we analyzed samples from British Columbia, Canada (WCAN) as well as a sample of the sister taxon *S. cariosus.* In the Atlantic, we analyzed samples from Maine (ME), Rhode Island (RI), Iceland (ICE), Norway (NOR), and the United Kingdom (UK). For all populations, we sequenced multiple libraries including: a single individual barnacle genome to ∼50× coverage, pools of 20–38 individuals per population (i.e., pool-seq; Schlotterer et al. 2014), as well as ∼600-bp amplicons from the mitochondrial (mtDNA) *COX I* gene (including previously published *COX I* data (Wares and Cunningham 2001)). We mapped these data sets to our newly assembled *S. balanoides* genome (supplementary appendix 1, Supplementary Material online) and characterized genetic diversity across all populations (supplementary appendix 2, Supplementary Material online). We first present our findings in the context of the barnacle’s phylogeography and demographic history. This is pivotal to understand the historical conditions which can contribute to ecological load. Then, we characterize the pervasiveness of balancing selection across the genome, as well as the age of balanced polymorphisms and their putative functional significance in highly heterogeneous environments.

## Results

### Standing Variation across Oceans

Our pool-seq panels discovered ∼3M high-quality single nucleotide polymorphisms (SNPs) across populations at common allele frequencies (>5%). When linkage is removed at 500 bp, the SNP panel thins to ∼690,000. Principal component analysis (PCA), on the Linkage disequilibrium (LD) thinned SNPs, shows that variation is strongly subdivided by ocean basins (fig. 1*D*). PC1 captures 74% of the variation and partitions populations across basins. PC2 (8.5% var.) partitions Atlantic populations into two discrete east–west clusters. The western cluster contains ME, RI, and ICE, and the eastern cluster contains UK and NOR. These clusters are supported by the abundance of mtDNA haplotypes within and between ocean basins (fig. 1*D* inset; supplementary table S1, Supplementary Material online) (Wares and Cunningham 2001; Flight et al. 2012; Nunez et al. 2018). The large divergence between oceans is also captured in levels of nucleotide diversity (*π*; a metric of standing genetic variation). Surprisingly, North Atlantic populations harbor more genetic variation (*π* = 1.05%) than their Pacific, ancestral, conspecifics (*π* = 0.55%; fig. 1*E*;supplementary fig. S1, Supplementary Material online). We also estimated the Tajima’s *D* statistic (*D*), a neutrality test based on the comparison of two measures of nucleotide polymorphism: *π* and Watterson theta (*θ*). The value of the *D* statistic is a good proxy for the excess (*D *<* *0), or deficit (*D *>* *0), of rare alleles in populations. These data indicate that all North Atlantic populations, especially NOR, have negatively skewed genome-wide values of *D* (fig. 1*F*, supplementary fig. S2, Supplementary Material online).

### Historical Phylogeography and Structure

We reconstructed changes of historical effective population sizes (*N*_e_) with the multiple sequentially Markovian coalescent model (MSMC) using individual whole genomes (Schiffels and Durbin 2014). Our results provide evidence for different phylogeographic trajectories in response to the events of the glaciations (fig. 1*G* and *H*). For instance, the Eastern Cluster and the Western Cluster populations shared a common demography throughout the Pleistocene (fig. 1*G*) but diverged in recent geological time. Namely, Eastern populations (especially NOR) experienced striking increases in *N*_e_ in the recent past (fig. 1*I*), likely following the asynchronous deglaciation of the Fennoscandian ice sheet (Ruddiman and Mcintyre 1981; Patton et al. 2017). Western populations, on the other hand, experienced a demographic contraction which started during the last glacial period and ended during the last glacial maxima (∼20 ka; fig. 1*J*) (Brochmann et al. 2003; Maggs et al. 2008; Flight et al. 2012).

We estimated gene flow by computing *f*_3_ statistics (Reich et al. 2009) for all possible combinations of target, source 1, and source 2 populations, using individual whole genomes (supplementary fig. S3 and table S2, Supplementary Material online). Our analysis finds no evidence of recent gene flow across oceans. This result is supported by two additional lines of evidence. First, a mtDNA molecular clock analysis (Drummond et al. 2002) suggests that Pacific and Atlantic populations have not exchanged migrants in nearly 2 My (supplementary appendix 3, Supplementary Material online); and second, estimates of genetic differentiation (*F*_ST_) reveal large amounts of genome-wide divergence (supplementary fig. S4, Supplementary Material online) and foreshadow the onset of allopatric speciation across oceans. Within the North Atlantic, *F*_ST_ is low (likely due to shared demography until the glacial maximum) and the *f*_3_ analysis suggests that admixture is pervasive (supplementary fig. S3 and table S2, Supplementary Material online). These findings are supported by additional ABBA–BABA tests for gene tree heterogeneity (Green et al. 2010) (see supplementary appendix 4, Supplementary Material online). Overall, these findings present three important points: First, they exemplify the complex demography that underlies standing variation in natural populations; second, they confirm that barnacles harbor high levels of genetic variation genome-wide; and third, they reveal the pervasiveness of gene flow and shared variation within ocean basins, where environmental heterogeneity is extensive across “micro” (1–3 m) and “meso” (1–10 km) scales. These conditions provide the environmental context for ecological load at the genomic scale.

### Balancing Selection in Barnacles

Balancing selection is expected to produce molecular and phylogenetic footprints not consistent with neutrality (Fijarczyk and Babik 2015). Molecular footprints include: enrichment of old alleles (e.g., trans-species polymorphisms; TSPs), elevated genetic variation (high *π*), deficit of rare alleles (*D *>* *0), excess SNPs at medium allele frequencies, reduced divergence around the balanced locus (low *F*_ST_), as well as the accumulation of nonsynonymous variation in the vicinity of balanced polymorphisms, a phenomenon known as sheltered load (Uyenoyama 2005). Likewise, balancing selection will produce a phylogenetic signal composed of diverged clades, corresponding to the balanced haplotypes. Deeply diverged clades will occur when balancing selection has maintained variation over long evolutionary times (i.e., ancestral balancing selection; Fijarczyk and Babik 2015). Notably, these signatures may become highly localized in the genome as the action of recombination over long periods of time will erode long-distance haplotype signatures.

**Fig. 2.**
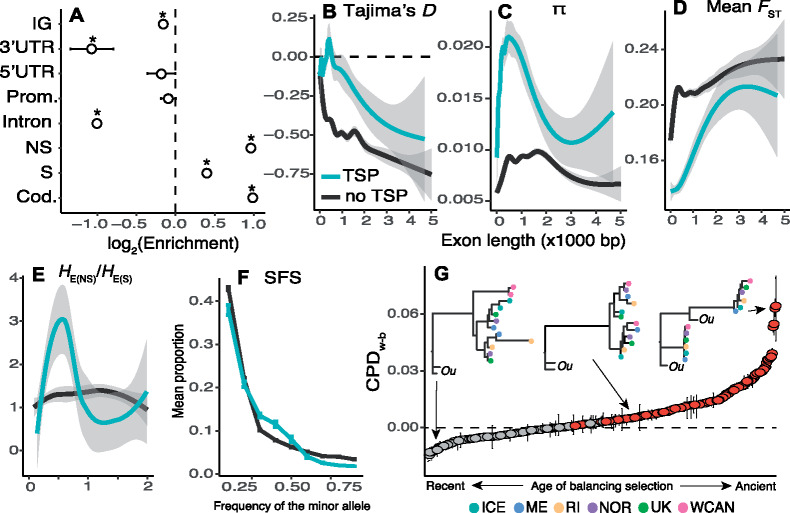
Evidence for balancing selection across the genome. (*A*) Enrichment analysis of TSPs across the genome of *Semibalanus balanoides* based on all populations studied. The asterisk symbols represent statistical significance. Prom., promoters; NS, nonsynonymous loci; S, synonymous loci; Cod., coding loci. (*B*) Plot of Tajima’s *D* (as a function of length) of exons bearing TSPs versus all other exons not bearing TSPs. (*C*) Same as (*B*) but for nucleotide diversity (*π*). (*D*) Same as (*B*) but for mean *F*_ST_. (*E*) Same as (*B*) but for the ratio of nonsynonymous heterozygosity to synonymous heterozygosity. (*F*) SFS for whole genes with TSPs versus other genes. Vertical bars are 95% confidence intervals. (*G*) Candidate genes under balancing selection ranked according to their CPD_w–b_ values (interquartile ranges shown as error bars). Red values indicate statistical significance. Horizontal dashed line indicates CPD_w–b_ = 0. In the *x*-axis, the label “ancient” refers to allele trees whose topology violates the genome-wide phylogeographic expectation (e.g., fig. 1*D*). “Recent” denotes the opposite case. Three example allele tree topologies are shown. The sister taxon, *Semibalanus cariosus*, is shown as “*Ou*” (for outgroup). The *x*-axis for (*B*), (*C*), (*D*), and (*E*) is exon length (×1,000 bp).

A joint analysis of our Pacific, Atlantic, and outgroup (*S. cariosus*) data sets reveal 11,917 cosmopolitan SNPs (i.e., SNPs that segregate in all populations across both oceans) which are also TSPs (supplementary data set S1, Supplementary Material online). TSPs, genome-wide, occur in 0.14% coding regions, 0.21% in introns, 0.02% in promoters, 0.01% in 5′-UTRs, and <0.01% in 3′-UTRs. The remainder of TSPs occurs in 0.09% of intergenic regions. An enrichment analysis which compares the abundance of TSPs, of each genomic class, relative to all discovered SNPs, reveals that TSPs are significantly overenriched in coding loci (fig. 2*A*), and 4,415 segregate at high frequencies in all populations (TSPs with heterozygosity [*H*_E_] > 0.30; supplementary fig. S5, Supplementary Material online). These patterns of variation could be the result of neutral processes such as recurrent mutation (homoplasy) across all populations of either species. However, the enrichment of cosmopolitan, nonsynonymous, TSPs at common frequencies is not consistent with neutrality. Under a model of strict neutrality, segregating mutations are eventually lost in populations after speciation (Clark 1997). Moreover, coding regions are subjected to purifying selection which removes deleterious and mildly deleterious nonsynonymous variants (Hartl and Clark 1997).

We compared patterns of genetic variation in exons bearing TSPs and other exons. When accounting for exon length, we observe consistently elevated values of *D* and *π* for TSP-bearing exons relative to other exons (fig. 2*B* and *C*; supplementary fig. S6, Supplementary Material online)*.* Except for the ME versus RI comparison (supplementary fig. S7, Supplementary Material online), TSP-bearing exons have consistently low *F*_ST_ values (fig. 2*D*). To quantify sheltered load, we compared the ratio of *H*_E_ values at nonsynonymous and synonymous mutations in TSP-bearing and other exons. Our results show that medium sized TSP-bearing exons (∼500 bp) harbor an excess of nonsynonymous *H*_E_ (fig. 2*E*). Notably, we observed that differences between TSP-bearing and other exons become less apparent as exons get longer. The relationship between exon size and the intensity of the balancing selection signatures depends on local recombination rates. Although exact recombination rates are not yet available for *Semibalanus*, empirical data suggest that LD decays at distances <1 kb (supplementary fig. S8, Supplementary Material online). As such, the signals of deviation from neutrality are more apparent on shorter exons, relative to longer ones. We observe 1,107 TSPs that cause nonsynonymous changes and occur in 312 genes with high-confidence annotations (4%; supplementary data set S2, Supplementary Material online). Consistent with our expectation of balancing selection, site frequency spectrum (SFS) analyses show that these 312 candidate genes harbor an excess of SNPs at medium allele frequencies relative to other annotated genes (fig. 2*F*).

### Age of Balanced Polymorphisms

To determine the age of the putatively balanced polymorphisms, we ran topological tests on the allele trees for each TSP region across the 312 candidate genes. We built trees using phased haplotypes for each TSP-bearing region for all single-individual genomes. We used these allele trees to compute the cophenetic distance (CPD) between tips. We classified allele trees as having or lacking highly diverged alleles based on the relative mean CPD between haplotypes from the same population versus from different populations (CPD_w–b_; see supplementary methods, Supplementary Material online). The analysis reveals that of the 312 allele trees, 150 carry a significant signature of ancestral balancing selection (CPD_w–b_ > 0, Bonferroni *P *<* *1 × 10^−9^; fig. 2*G*;supplementary data set S2, Supplementary Material online). This suggests maintenance of diverged haplotypes for more than 2 My, with extreme cases in which haplotypes are shared across species (8–10 My) (Perez-Losada et al. 2008; Herrera et al. 2015). The remaining genes with CPD_w–b_ <0 may represent either cases where the balanced alleles are younger or oversampling of homozygous individuals for any given marker.

### Targets of Selection

**Fig. 3.**
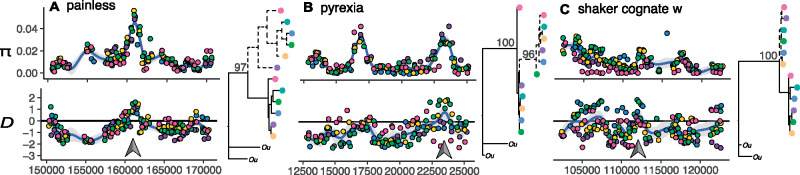
Balancing selection on ecologically important genes. We present patterns of genetic variation (*π* and *D* estimated from pool-seq data, and allele tree topologies estimated from single individuals) for three example genes: (*A*) painless (*Pain*), (*B*) pyrexia (*Pyx*), and (*C*) shaker cognate w (*Shaw*). Gray arrows show regions that contain TSPs. In Tajima’s *D* panels, the horizontal line marks the *D* = 0 point. For all trees, the sister taxon, *Semibalanus cariosus*, is shown as “*Ou*.” The colors represent populations: WCAN (pink), ME (blue), RI (yellow), ICE (dark green), NOR (purple), UK (light green). The *x*-axis shows base pair position within scaffolds.

We partitioned our data set among candidate genes with positive and negative CPD_w–d_ allele trees and conducted gene ontology (GO) enrichment analyses. The 150 genes with positive CPD_w–d_ trees show enrichment for terms related to “ion channel regulation,” including genes involved in environmental sensing, and circadian rhythm regulation (supplementary table S3, Supplementary Material online). We show examples for three candidate genes under ancestral balancing selection involved in environmental sensing: 1) the painless gene (*Pain*; g1606; fig. 3*A*), which is involved in nociception (i.e., pain reception), as well as detection of heat and mechanical stimuli (Tracey et al. 2003; Xu et al. 2006); 2) the Pyrexia gene (*Pyx*; g3472; fig. 3*B*), which is involved in negative geotaxis, and responses to heat (Lee et al. 2005); and 3) the shaker cognate w gene (*Shaw*; g3310; fig. 3*C*), which is involved in regulation of circadian rhythm (Hodge and Stanewsky 2008; Buhl et al. 2016). These three examples showcase canonical footprints of balancing selection around the TSP, concomitant with a bimodal allele tree. Among genes with negative CPD_w–d_, we observe enriched functions for “anatomical structure formation” including genes coding for motor proteins and muscle genes (supplementary table S4, Supplementary Material online). In all cases, we used RNA-seq data from ME individuals to confirm that these loci are expressed in adult barnacles.

## Discussion

In intertidal barnacles, the dichotomy of strong adult selection and high offspring dispersal means that any allele that is beneficial to parental fitness in one generation may be neutral or deleterious in the next (Gillespie 1973). This leads to a fundamental question in evolutionary biology: How are adaptations maintained in the face of extreme ecological variability? In this article, we provide evidence suggesting that balancing selection is widespread across the barnacle genome, with 4% of annotated genes harboring putatively balanced polymorphisms. Notably, these polymorphisms occur in genes with functions that may be important for life in variable environments, and many have been maintained for at least 2 My despite a complex phylogeographic history (Wares and Cunningham 2001; Flight and Rand 2012). Naturally, the heterogeneous nature of the rocky intertidal imposes a segregation “cost” for these balanced polymorphisms, as they occur in individuals that, due to high dispersal, recruit in suboptimal habitats for any given genetic makeup. This ecological load, defined as $L_{e}=\;(W_{\max}-\;\overset{-}{W})/{\;W}_{\max}$ (where $\overset{-}{W}\;$is mean fitness, and$\;W_{\max}$ is optimal fitness, across all habitats), will be substantial, as demonstrated by comprehensive recruitment studies in natural habitats (Bertness 1989; Bertness et al. 1992; Pineda et al. 2006). For example, at initial settlement, barnacle density can be as high as 76 individuals per cm^2^, but at maturity, it can be as low as 0.15 individuals per cm^2^ (0.2% survival) (Pineda et al. 2006). This mass mortality is habitat- and genotype dependent (Schmidt and Rand 2001). This is the type of “fitness cost” envisioned in the Levene model of balancing selection (Levene 1953). As such, our data suggest that the problem of ecological load is a defining condition of the barnacle life cycle. And, more generally, it argues that balancing selection, via marginal overdominance, may be the fundamental process underlying maintenance of adaptation in variable environments.

### Is Pervasive Balancing Selection Plausible in Nature?

Under classical models of population genetics, when loci are considered to be independent of each other, the additive effects of widespread balanced polymorphism result in unbearable amounts of fitness variance and genetic death (Kimura and Crow 1964; Lewontin and Hubby 1966). However, if balanced loci have interactive effects (e.g., epistasis), multiple polymorphisms could be maintained with minimum effects on the distribution of fitness variance (King 1967; Milkman 1967; Sved et al. 1967; Wittmann et al. 2017). Based on this theoretical framework, multiple models have been developed to describe the conditions that favor the long-term maintenance of functional variation in spatially varying environments (Gillespie 1973; Hedrick et al. 1976). Moreover, polymorphisms will be less likely to be lost if there is a large number of ecological niches available, if there is migration among niches, and if individuals are proactive in choosing niches where their fitness is maximized (Hedrick et al. 1976). We argue that barnacles satisfy these conditions to some degree.

First, although it is useful to summarize intertidal heterogeneity in the form of discrete microhabitats (Schmidt et al. 2000), individual barnacles experience the rocky shore as a complex tapestry of interactive stressors at three spatial levels. At microhabitats scales, the upper and lower tidal zones pose diametrically different ecological challenges in terms of food availability, competition, predation, and risk of desiccation (Bertness et al. 1991; Schmidt and Rand 1999, 2001). At mesohabitat scales, open coasts versus sheltered estuaries vary in their exposure to wave action, upwelling dynamics, and biotic interactions (Sanford and Menge 2001; Dufresne et al. 2002; Veliz et al. 2004). These, in turn, modify microlevel stressors. Lastly, at macrohabitat scales, topological differences across shores and latitudinal variations in tidal range produce a mosaic of thermal stress along continents (Helmuth et al. 2002). Consequentially, what selection pressures are more important for any given barnacle will emerge from the interactions among these stress gradients. This complex landscape of selection has been captured in studies of the barnacle *Mpi* gene. Accordingly, the locus is under selection at microlevels in the Gulf of Maine (Schmidt and Rand 1999; Schmidt et al. 2000), at mesolevels in the gulf of St Lawrence (Canada) (Dufresne et al. 2002; Veliz et al. 2004), yet it shows tepid signs of selection in the Narragansett Bay (Rhode Island) (Rand et al. 2002; Nunez et al. 2020). Similar complexity has also been captured in temperate populations of *Drosophila.* In these, idiosyncratic weather effects can alter the dynamics of seasonal adaptation (Bergland et al. 2014; Machado et al. 2019). Second, the high dispersal capacity of the larval stage ensures constant migration between these niches across generations. Finally, barnacles also have the ability to choose preferred substrates during settlement. This occurs during the spring when barnacle larvae extensively survey microhabitats for biological, chemical, and physical cues produced by previous settlers before making final commitments of where to settle (Bertness et al. 1992). Unfortunately for the barnacle, this capacity for substrate choice does not mitigate mass mortality during late summer, which leads to strong selection for particular genotypes (Schmidt and Rand 2001). Currently, there is limited evidence for genotype-specific substrate selection or nonrandom settlement (Veliz et al. 2006). A cohort-tracking and sequencing experiment could be utilized to address this question (these experiments are underway). If true, these behaviors may constitute a form of adaptive plasticity, helping barnacles choose habitats where their fitness may be marginally improved. Overall, this suggests that the barnacle’s life history is conducive to the maintenance of balanced polymorphisms.

### Pool-seq in Ecological Genomics

Our analysis was conducted using pool-seq data. As such, it is important to highlight known caveats associated with this genotyping technology (Anderson et al. 2014; Anand et al. 2016; Nunez et al. 2018). Although incredibly cost-effective, the accuracy of pool-seq is highly dependent on the number of individuals pooled, sequencing coverage, as well as sequencing technology. These caveats can become pronounced when working on nonmodel systems where enforcing uniform sample sizes across populations may be logistically challenging. Nevertheless, pool-seq experiments that deviate from the recommended design (Gautier et al. 2013) result in inaccurate estimates of allele frequency, including undersampling of rare alleles and oversampling of fixed sites (Anderson et al. 2014). These systemic errors have notable impacts when estimating demographic parameters. We ameliorated these shortcomings using a two-pronged approach. First, for each population sampled, we sequenced both a single individual and a pool. The single individual allowed us to estimate demographic parameters. The pool, on the other hand, allowed us to survey common variation across populations. And thus, although each individual approach has unique shortcomings, their combination provides a robust data set to address the questions presented in this study. In addition, because one must filter out sequencing errors, most implementations of high-throughput sequencing in ecological genomics produce skewed SFS distributions by undersampling low-frequency mutations (Achaz 2008, 2009). This problem is exacerbated for pool-seq experiments and can produce biased estimates of common statistics such as *θ* and, consequently, Tajima’s *D*. However, because we are interested in understanding patterns of genetic variation at common variants, our analyses are less susceptible to this drawback.

### What Variation Is under Selection?

Our analyses suggest that 4% (312) of all annotated genes are candidates of balancing selection across the entire range of the species. Although follow-up experiments are needed to determine the replicability and functional importance of these variants, our evidence for balancing selection is consistent with patterns reported for other species. For example, the number of candidate genes in *Semibalanus* is like that observed in *Arabidopsis thaliana* and its close relative *Capsella rubella* (433 genes) (Wu et al. 2017). Similar to *Semibalanus*, these plants diverged ∼8 Ma, and their natural populations experience high levels of ecological heterogeneity (Bakker et al. 2006). We must acknowledge that our number may be an underestimation driven by the nascent state of the genomic tools in *Semibalanus*. Future genome assemblies, combined with improved annotations, will undoubtedly yield a more complete picture of functional variation in the species. In addition, it will allow for a more comprehensive characterization of selection in structural variants and regulatory loci, which have been shown to be fundamental in the evolution of complex phenotypes (Wray 2007; Faria et al. 2019). Despite these limitations, our analysis recovered many candidate genes involved in functions which may be key for life in variable environments. Without more functional validation, the connections between these genes and barnacle ecology are merely speculative. However, many of these candidates have been studied in other systems in the context of stress tolerance. Consequentially, they are fertile grounds for hypothesis generation and follow-up experiments. For instance, the general enrichment for ion channel genes suggests selection related to osmotic regulation (Sundell et al. 2019). This hypothesis is highly plausible given that intertidal ecosystems experience strong salinity fluctuations, repeatedly exposing barnacles to osmotic challenges at all spatial scales. In addition, we observe targets of selection involved in environmental sensing loci (e.g., *pain*, *pyx*, and *shaw;*fig. 3). Similar to osmotic regulation, selection on these genes is entirely plausible given the inherent variability of intertidal habitats. An important hypothesis from the allozyme era is the idea that balancing selection would target genes at the node of metabolic fluxes (Eanes 1999; Watt and Dean 2000). In such cases, balanced variation would provide biochemical flexibility to cope with environmental heterogeneity. In the same vein, we hypothesize that balancing selection may act more often on “sensor genes” which control plastic responses to ecological variation. Testing this hypothesis is beyond the scope of this study and would require the use of allele-specific differential expression experiments in barnacles. We also note that evidence of balancing selection and TSPs at the *Mpi* gene are discussed in Nunez et al. (2020).

### Complex Demography and Speciation

Our demographic analyses provide clues about how historical events affected genetic variation in barnacle populations. In the Atlantic, our evidence suggests a shared demography throughout the Pleistocene, and that the modern Eastern and Western clusters formed in response to recent events of the last glacial cycle. These findings highlight that the low *F*_ST_ values observed within the basins arise due to shared ancestry. Moreover, they also suggest that population structure persists in the presence of gene flow. As such, although larvae have the capacity to disperse for hundreds of kilometers, ocean currents (Nunez et al. 2018) and different estuarine flushing times (Brown et al. 2001) allow regions to retain some level of geographical structuring (Johannesson et al. 2018; Nunez et al. 2018). Comparisons between oceans reveal a stark pattern of genome-wide divergence. This pattern is driven by the separation of Pacific and Atlantic populations following the events of the trans-Arctic interchange (Vermeij 1991). Accordingly, the negative levels of *D* in the North Atlantic may reflect the effect of bottlenecks during the trans-Arctic interchange. Notably, the high levels of *π* in the Atlantic are not concordant with predictions of common colonization models in which variation of the younger population is a subset of the ancestral population (Maggs et al. 2008). We hypothesize that this could be the result of ancient admixture due to repeated trans-Arctic invasions from the Pacific (Väinölä 2003). We recognize that ancestral admixture could generate artificial signatures of balancing selection via the mixing of highly differentiated haplotypes. However, such an occurrence would affect most genes in the genome. Our evidence shows that the signatures of balancing selection are highly localized in TSP regions. For example, although *D* is elevated in TSP regions, it is negatively skewed genome-wide. Our data do not support recent gene flow between ocean basins. As such, after 2 My of separation, neutral divergence appears to be driving Atlantic and Pacific populations to speciate in allopatry. A closer look to this hypothesis will require crossing individuals from both basins, and surveying offspring fitness and viability. More salient, however, is the observation of shared haplotypes between oceans in our candidate genes for balancing selection. In light of such strong background divergence, this provides evidence that balancing selection on most of these genes is strong and that polymorphisms have been maintained for long periods of time.

## Materials and Methods

### Barnacle Collections

Barnacle samples were collected from Damariscotta (Maine, United States; ME), Jamestown (Rhode Island, United States; RI), Calvert Island (British Columbia, Canada; WCAN), Reykjavik (Iceland; ICE), Porthcawl (Wales, United Kingdom; UK), and Norddal (Norway; NOR). Additional samples were collected in Bergen (Norway), Tórshavn (Faroe Island), and Tjärnö (Sweden). For all samples, species identities were confirmed using Sanger sequencing of the mtDNA COX I region (Bucklin et al. 2011). For the WCAN, RI, ME, ICE, UK, and NOR populations, we collected a single individual for DNA-seq, and a group of 20–40 individuals for pool-seq (supplementary appendix 2, Supplementary Material online). RNA-seq was done on four individuals from Maine. DNA-seq was done on a single individual from the sister taxa *S. cariosus*. DNA/RNA was extracted using Qiagen DNeasy/RNeasy kits. All pools and single individuals were sequenced in their own lanes of an Illumina machine by GENEWIZ LLC using 2 × 150 paired-end configuration.

### Mapping Data Sets to the Genome

Samples were mapped to a genome assembled de novo for the species (Sbal3.1; NCBI GenBank accession: VOPJ00000000; BioProject: PRJNA557548; BioSample: SAMN12406453; supplementary appendix 1, Supplementary Material online). The genome was assembled using a hybrid approach which combines PacBio reads and Illumina reads using DBG2OLC (Ye et al. 2016) and Redundans (Pryszcz and Gabaldon 2016). Gene models were constructed using an ab initio method, AUGUSTUS (Stanke and Waack 2003), informed by evidence from the RNA-seq. A gene feature file (GFF) is available as supplementary data set S4, Supplementary Material online. The model used for gene prediction was trained in *Drosophila melanogaster*. Genes were annotated by pairwise blast against the *D. melanogaster* genome (Dmel6; NCBI GenBank: GCA_000001215.4). All annotations are available as supplementary data set S5, Supplementary Material online. DNA reads from all populations were mapped to Sbal3.1 using bwa mem (Li 2013). RNA reads were mapped using HiSat2 (Kim et al. 2015). SNPs were called using the samtools pipeline (Li et al. 2009). Short-read phasing was done using HAPCUT2 (Edge et al. 2017). LD in pools was estimated using LDx (Feder et al. 2012).

### Genome Analyses

Estimates of *π* and *D* were done using the popoolation-1 suite (Kofler, Orozco-terWengel, et al. 2011). Estimations of allele frequencies and *F*_ST_ were done using the popoolation-2 suite (Kofler, Pandey, et al. 2011). Demographic reconstructions were done using MSMC (Schiffels and Durbin 2014). The *f*_3_ statistics were estimated using treemix (Pickrell and Pritchard 2012). Bayesian molecular clock analyses were done in BEAST2 (Bouckaert et al. 2014). ABBA/BABA statistics were calculated in *Dsuite* (Malinsky et al. 2020). Phylogenetic inferences were done in iQtree (Chernomor et al. 2016). GO enrichment analysis was done using GOrilla (Eden et al. 2009) and GO terms inferred from our *Drosophila* annotation. The enrichment was assessed by comparing two genes list. The first composed of the genes of interest (i.e., the gene targets), the second one by all the genes annotated in Sbal3.1 (i.e., the gene universe). A detailed description of our analyses can be found in the supplementary methods section, Supplementary Material online, as well as in GitHub: https://github.com/Jcbnunez/BarnacleEcoGenomics.

## Supplementary Material

Supplementary data are available at *Molecular Biology and Evolution* online.

## Supplementary Material

msaa227_Supplementary_Appendix
